# Effect of Stimulus Contrast and Visual Attention on Spike-Gamma Phase Relationship in Macaque Primary Visual Cortex

**DOI:** 10.3389/fncom.2018.00066

**Published:** 2018-08-14

**Authors:** Aritra Das, Supratim Ray

**Affiliations:** Centre for Neuroscience, Indian Institute of Science, Bangalore, India

**Keywords:** attention, spike-field coherence, spike-gamma phase, contrast, area V1, stLFP

## Abstract

Brain signals often show rhythmic activity in the so-called gamma range (30–80 Hz), whose magnitude and center frequency are modulated by properties of the visual stimulus such as size and contrast, as well as by cognitive processes such as attention. How gamma rhythm can potentially influence cortical processing remains unclear; previous studies have proposed a scheme called phase coding, in which the intensity of the incoming stimulus is coded in the position of the spike relative to the rhythm. Using chronically implanted microelectrode arrays in the primary visual cortex (area V1) of macaques engaged in an attention task while presenting stimuli of varying contrasts, we tested whether the phase of the gamma rhythm relative to spikes varied as a function of stimulus contrast and attentional state. A previous study had found no evidence of gamma phase coding for either contrast or attention in V1, but in that study spikes and local field potential (LFP) were recorded from the same electrode, due to which spike-gamma phase estimation could have been biased. Further, the filtering operation to obtain LFP could also have biased the gamma phase. By analyzing spikes and LFP from different electrodes, we found a weak but significant effect of attention, but not stimulus contrast, on gamma phase relative to spikes. The results remained consistent even after correcting the filter induced lags, although the absolute magnitude of gamma phase shifted by up to ~15°. Although we found a significant effect of attention, we argue that a small magnitude of phase shift as well as the dependence of phase angles on gamma power and center frequency limits a potential role of gamma in phase coding in V1.

## Introduction

Gamma oscillations are rhythmic fluctuations in a frequency range between 30 and 80 Hz in brain signals (Buzsaki, 2006; Buzsáki et al., 2013), which have been consistently linked with high-level cognitive processes such as attention (Fries et al., 2001; Gregoriou et al., 2009), perception (Rodriguez et al., 1999) and feature binding (Singer, 1999). In recordings from the primary visual cortex (area V1), gamma is also known to be highly dependent on the properties of visual stimulus, such as size (Gieselmann and Thiele, 2008; Ray and Maunsell, 2011a; Jia et al., 2013), orientation (Berens et al., 2008; Jia et al., 2011), and contrast (Ray and Maunsell, 2010; Jia et al., 2013). Although several hypotheses about how gamma rhythm could influence neural processing have been proposed, such as binding by synchrony (Singer, 1999) and communication-through-coherence (Fries, 2015), whether gamma plays a functional role remains unclear (Ray and Maunsell, 2015).

Here we test a specific hypothesis called phase coding (PC), originally proposed in the context of theta rhythm in the hippocampus (O'Keefe and Recce, 1993; Buzsáki and Chrobak, 1995), in which information is coded in the position of the spike relative to the rhythm. In the context of gamma rhythm (Fries et al., 2007), which is thought to be associated with an inhibitory network of interneurons (Bartos et al., 2007; Cardin et al., 2009; Sohal et al., 2009), this hypothesis posits that the rhythmic network inhibition interacts with excitatory input to pyramidal cells such that the more excited cells (which can overcome the inhibition earlier) fire earlier in the gamma cycle. Thus, stimulus intensity can be coded in the gamma phase relative to the spike. However, whether gamma PC occurs is controversial, with evidence both in favor and against the hypothesis. We have earlier shown that in macaque secondary somatosensory cortex, the phase of gamma rhythm does not vary with stimulus intensity (Ray et al., 2008). In V1, one study showed some evidence of PC with different orientations (which they took as a proxy for stimulus intensity), at least for sites that had weak gamma power and weak gamma-spike phase locking (Vinck et al., 2010). Other studies in V1 showed no evidence of PC when the stimulus contrast (a more direct index of stimulus intensity as compared to orientation) was varied (Chalk et al., 2010; Ray and Maunsell, 2010). Importantly, Chalk and colleagues further showed that even attention, which increases the effective contrast of the stimulus (Carrasco et al., 2004), does not cause a shift in spike-gamma phase. They also showed that in V1, attention causes a reduction in gamma power and spike-gamma coupling (Chalk et al., 2010), exactly opposite of what has been shown in higher cortical areas such as V4 (Fries et al., 2001).

While Chalk et al. (2010) failed to provide evidence in favor of PC, one limitation of their study was that spikes and local field potential (LFP) were collected from the same electrode, which can potentially bias the spike-gamma phase relationship because of the presence of spike-related transients (Ray, 2015). Specifically, spikes are associated with a “transient” in the LFP recorded from the same electrode, which could be due to synaptic activity that leads to the spike as well as low-frequency component of the action potential (spike “bleed-through”; see Ray, 2015, for details). The remaining studies either removed this transient using signal processing techniques such as Matching Pursuit (Ray et al., 2008), or used spikes and LFP from different electrodes (Ray and Maunsell, 2010; Vinck et al., 2010) that reduces the bias (see section Discussion for more details on this), but none of these reports studied the effect of attention on spike-gamma phase. To test whether stimulus contrast can be coded in the phase of the gamma rhythm, we here trained monkeys to do a demanding attention task while presenting stimuli that varied in contrast to study the effect of both contrast and attention on spike-gamma phase, while recording from chronically implanted microelectrode arrays such that spike-gamma phase could be estimated using spikes and LFPs recorded from different electrodes. Note that since attention is thought to increase the effective stimulus contrast (Carrasco et al., 2004), testing whether gamma phase varies with attentional state is also a test for gamma PC for contrast. We further studied the effect of the online causal filter used to obtain the LFP, which introduces a delay in the LFP and has been shown to influence spike-LFP relationships (Okun, 2017), but has not been accounted for in previous studies.

## Materials and methods

Experimental procedures have been described in detail in earlier studies (Ray and Maunsell, 2010, 2011b; Shirhatti et al., 2016); we provide a brief description here.

### Ethics statement

The animal protocols reported in this study were approved by the Institutional Animal Care and Use Committee of Harvard Medical School.

### Electrophysiological recordings

Two male rhesus monkeys *(Macaca mulatta)* were implanted with a scleral search coil and a head post and were subsequently trained to perform an attentionally demanding task. Once they learned the behavioral task, a microelectrode array (Blackrock Microsystems, 96 active electrodes) was implanted in the right V1 cortex (about 15 mm anterior from the occipital ridge and 15 mm lateral from the midline). The microelectrodes were 1 mm long and 400 μm apart from each other, with impedance between 0.3 and 1 MΩ at 1 kHz. Although histological analysis had not been performed to identify the exact location of the microelectrode tips, they are expected to be in cortical layer 2/3 or 4 based on the approximate thickness of V1 (2 mm; Hubel and Wiesel, 1977). Electrical signals were recorded using commercial hardware and software (Blackrock Microsystems), referenced to a wire placed on the dura near the microelectrode grid. Raw electrical signals were filtered between 0.3 Hz (Butterworth filter, 1st order, analog) and 500 Hz (Butterworth, 4th order, digital) and digitized at 2 kHz (16-bit resolution) to get the LFP. Multi-units were extracted by filtering the raw signal between 250 Hz (Butterworth filter, fourth order, digital) and 7,500 Hz (Butterworth filter, third order, analog) followed by an amplitude threshold (set at ~6.25 and ~4.25 of the signal SD for the two monkeys). To improve the quality of unit isolation, multi-units were subsequently sorted offline (Offline Sorter, Plexon Inc.). The receptive fields, obtained by flashing small Gabor stimuli on a rectangular grid that encompassed the receptive fields of all the electrodes in the array, were located in the lower left quadrant of the visual space at an eccentricity of about 3–5°. As in previous studies, only electrodes for which stable estimates of the receptive fields could be obtained (27 and 66 electrodes for the two monkeys), were used for subsequent analysis.

### Behavioral task paradigm

**Figure 1:**
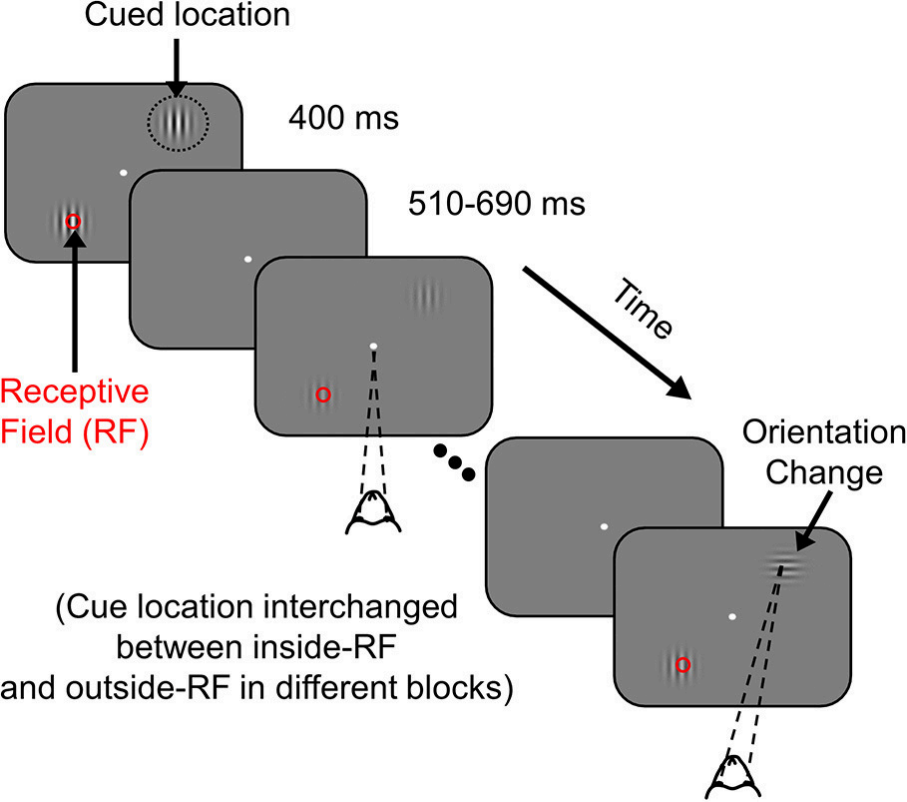
Attention Task. While the monkey maintained fixation, two achromatic Gabor stimuli were flashed for 400 ms with an inter-stimulus delay of 510–690 ms at two spatial locations from the fixation spot; one of the stimuli overlapped with the receptive field of recorded V1 neurons (indicated by a red circle for clarity; not visible to the monkey) and the other stimulus appeared at a location of equal eccentricity in the opposite hemifield. The monkey was cued to covertly attend to one of the two locations in different blocks of trials (indicated by black dotted circle, not visible to the monkey). At an unsignaled time, during one of the stimulus presentations, the orientation of the cued stimulus was changed by 90°. The monkey was rewarded with a drop of juice for making a saccade to the location of orientation change. If there was no change during a trial (catch trial), the monkey was rewarded for maintaining fixation throughout that trial.

The monkeys were required to maintain their gaze within 1° of a small central dot (0.05°-0.10° diameter) during the task while two achromatic odd-symmetric static Gabor stimuli were synchronously flashed for 400 ms with a mean inter-stimulus period of 600 ms. One of the two Gabor stimuli was centered on the receptive field of one of the recorded sites (new location for every session) while the second stimulus was located at an equal eccentricity on the opposite side of the central fixation point. The monkeys were cued to pay attention to one of the two stimulus locations in different blocks of trials by presenting two instruction trials (not included in the analysis) at the start of the block, in which there was only a single stimulus. The contrasts of the attended and unattended Gabor stimuli were equal on each presentation and could take any of the eight possible values: 0, 1.6, 3.1, 6.2, 12.5, 25, 50, and 100%, chosen pseudo-randomly. At an unstipulated time drawn from an exponential distribution (mean 2,000 ms, range 1,000–7,000 ms for Monkey 1; mean 3,000 ms, range 1,000–7,000 ms for Monkey 2), the orientation of the stimulus at the cued location changed by 90°. An exponential distribution was used to minimize expectation of target appearance and to keep the attentional state uniform during a trial since the hazard function is flat for an exponentially distributed target onset time. The monkeys were rewarded with a drop of juice for making a saccade to the location of the altered stimulus within 500 ms of orientation change. To account for saccade latency and to minimize guessing, monkeys were rewarded only for saccades beginning at least 100 ms after the orientation change. Trials were terminated at 7,000 ms if the target had not appeared, in which case the monkeys were rewarded for maintaining fixation throughout that trial. These catch trials were excluded from analysis (for more details, see Ray and Maunsell, 2010 and Figure 1).

The Gabor stimuli used for this task were both static with SD of 0.5°, spatial frequency of 4 cycles per degree, with one of the Gabor stimuli located at the center of the receptive field of one of the recorded sites (new recording site for each session), at its preferred orientation. Data from the two monkeys were collected in 10 and 17 recording sessions, respectively. Only correct trials were used for analysis. For each of the correct trials, only the second stimulus up to the last stimulus before target onset were used for analysis. We only used stimulus contrasts for which salient gamma oscillations were observed (25, 50, and 100% contrasts). For each contrast and attention condition, on average we obtained 79 ± 4 (range 55–101) stimulus repeats for Monkey 1 and 74 ± 5 (range 47–120) for Monkey 2.

### Electrodes and electrode pair selection

Electrodes with receptive field centers within 0.2° of the stimulus center in each of the recording sessions were used for analysis, yielding 63 electrodes (23 unique; many electrodes were selected in several recording sessions) for Monkey 1 and 89 electrodes (53 unique) for Monkey 2. These are referred to as “LFP” electrodes. For spike-field coherence (SFC), spike-triggered LFP (stLFP) and spike-gamma phase histograms, we selected a subset of the LFP electrodes from which at least 20 spikes could be recorded in the analysis interval (150–400 ms after stimulus onset) and the signal to noise ratio of the isolation (Kelly et al., 2007) was greater than 2. This generated 23 (12 unique) and 39 (27 unique) “spike” electrodes for Monkeys 1 and 2, respectively. For each session, we took all combinations of spike and LFP electrodes with receptive fields within 0.2° of the stimulus center, yielding 23 (12 unique) and 39 (27 unique) “same” spike-LFP pairs (**Figure 3**), and 163 (120 unique) and 170 (147 unique) pairs of “different” spike-LFP electrodes for Monkeys 1 and 2, respectively (**Figures 4**, **5**).

## Data analysis

All data were analyzed using custom codes written in MATLAB (The MathWorks, RRID: SCR_001622). Individual data analysis methods are briefly summarized below.

### Change in power spectral density (PSD) plots (Figure 2)

Stimulus-induced responses were first obtained by subtracting the mean LFP across all stimulus repeats for each condition (i.e., the event-related potential) from individual single trial time series data. Subsequent analyses were performed on these stimulus-induced responses. Power spectral densities (PSDs) for different stimulus and attention conditions were computed using the multi-taper method with 5 tapers using the Chronux toolbox (Bokil et al., 2010); http://chronux.org/, (RRID: SCR_005547). The analysis period was selected between 150 and 400 ms after stimulus onset to avoid stimulus onset related transients and compared against a “baseline period” between −300 and −50 ms of stimulus onset. To ensure that the change in power from baseline was not affected due to differences in the baseline power for different attention conditions, change in PSDs were plotted with respect to the baseline response of “attend-out” (attention directed outside the receptive field) condition for each stimulus contrast value:

$$\Delta PSD_{i}=10\left(\log_{10}{\;(ST)}_{i}-\log_{10}{\;(BL_{Att\;Out})}_{i}\right)$$

Here *i* represents the contrast condition (25, 50, or 100%), Δ*PSD*_*i*_ represents the change in PSD in decibels, (ST)_*i*_ denotes the PSD in the stimulus epoch and (*B*_*L*_*Att Out*_)*i*_ denotes the baseline PSD for attend-out condition.

**Figure 2:**
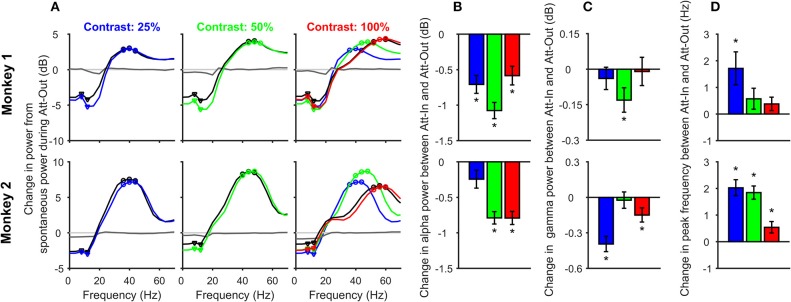
Change in alpha and gamma power and gamma peak frequency with contrast and attention. **(A)** Change in Power-spectral densities (PSDs) during stimulus period (150–400 ms after stimulus onset) from the pre-stimulus baseline (−300 to −50 ms) for different contrasts: 25% (left panel), 50% (middle), and 100% (right), for attend-in (colored) and attend-out (black) conditions, for 63 electrodes in Monkey 1 (top row) and 89 electrodes in Monkey 2 (bottom row). All changes were computed with respect to the baseline PSD during the attend-out condition. Also shown is the change in baseline PSD for attend-in condition (dark gray trace); the same for the attend-out case is trivially zero (light gray). PSD traces for 25 and 50% contrast for attend-in condition is overlaid (dashed-dot color traces) on the 100% contrast panel to show how gamma peak frequency changes with contrast. The alpha and gamma band frequencies used for subsequent analysis are shown in triangles and circles respectively. **(B)** Change in alpha power (in decibels) between attend-in and attend-out condition for 25% (blue), 50% (green), and 100% (red) contrasts. Error bars represent standard error of mean across 63 electrodes from Monkey 1 (top row) and 89 electrodes from Monkey 2 (bottom row). Significant differences (*p* < 0.05, *t*-test) are indicated by “*”. **(C)** Same as **(B)**, but for gamma power. **(D)** Change in gamma peak frequency (Hz) between attend-in and attend-out condition for contrasts 25% (blue), 50% (green), and 100% (red). Error bars represent standard error of mean across 63 electrodes from Monkey 1 (top row) and 89 electrodes from Monkey 2 (bottom row).

For the change in alpha power shown in Figure 2B, we first averaged the power between 8 and 12 Hz (triangles in Figure 2A; note that because we used an analysis interval of 250 ms, we had a frequency resolution of 4 Hz) and subsequently took the difference between the “attend-in” (attention directed inside the receptive field) and attend-out power on a log scale:

$$\Delta Power_{i}=10\left(\log_{10}{\;(ST_{Att\;In})}_{i}-\log_{10}{\;(ST_{Att\;Out})}_{i}\right)$$

Here (ST) _*i*_ denotes the alpha power in the stimulus epoch for the *i*th contrast condition. For gamma power, the same procedure was used with three frequency bins centered around the peak gamma frequency (shown in circles in Figure 2A). For computing peak gamma frequency, we choose the frequency bin for which Δ*PSD*_*i*_ attained its maximum value between 30 and 60 Hz.

### Coherency analysis (Figures 3–5)

The coherency between two signals *x* and *y* is computed using the following equation:

$$\begin{array}{l} Coherency_{xy}\;(f)=\frac{S_{xy}\left(f\right)}{\sqrt{S_{xx}\left(f\right)S_{yy}\left(f\right)}} \end{array}$$

Where *S*_*xy*_ (*f*) denotes the cross-spectrum between the signals *x* and *y* and *S*_*xx*_ (*f*) and *S*_*yy*_ (*f*) denote the auto spectra of each signal. The coherency values were computed using the multi-taper method implemented in Chronux toolbox using five tapers. All the coherence analyses were performed using the sorted multiunit dataset. For spike-field coherence, the spike time series was converted to a binary time series (at 0.5 ms resolution) with a “1” at each time position containing a spike and “0” otherwise (500 data points for the stimulus period). The results were similar for three tapers. All the circular statistical analyses were performed using an open source circular statistics toolbox in MATLAB (CircStat; Berens, 2009). Spike-triggered LFP (stLFP) were computed by taking a ±25 ms segment of the LFP around each spike in the stimulus period and subsequently taking the average of those segments.

**Figure 3:**
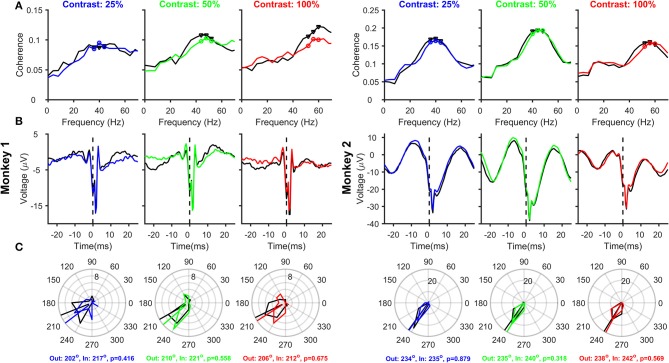
Relationship between Spikes and LFPs recorded from the same electrode, as a function of Contrast and Attention. **(A)** Mean spike-LFP coherence for 25, 50, and 100% contrasts for Monkey 1 (three columns on the left) and Monkey 2 (three columns on the right). Black and colored traces represent attend-out and attend-in conditions respectively. Spike-LFP coherence was computed for the time interval between 150 and 400 ms post stimulus onset. Spikes and LFP were recorded from 23 and 39 electrodes in the two monkeys, whose receptive fields were within 0.2° of the stimulus center. **(B)** Average spike-triggered LFP average for the same electrode and analysis duration as **(A)**; Time of the spike (0 ms) is shown by a dotted line for clarity. **(C)** Phase histograms of the spike-LFP coherence values at peak and two surrounding gamma frequencies, for attend-out condition (indicated by black triangles in each panel in **(A)** and attend-in condition (indicated by colored circles in each panel in **(A)**. The circular means of the spike-gamma phase values are indicated for attend-out and attend-in condition, along with the *p*-value obtained from Watson-Williams test to compare the mean phases.

### Removing filtering effect (Figure 5)

**Figure 4:**
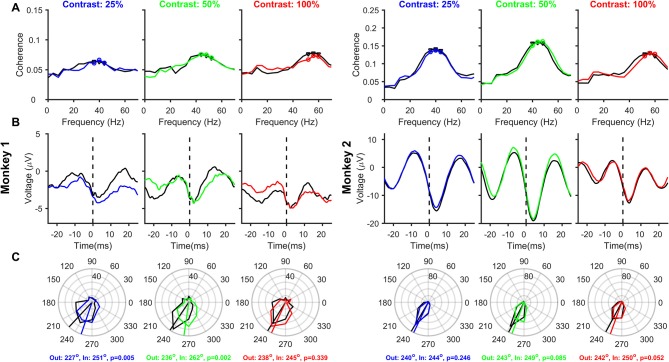
Relationship between Spikes and LFPs recorded from different electrodes, as a function of Contrast and Attention. Same as Figure 3, but the analysis is performed on 163 and 170 pairs of different spike-LFP electrodes (the receptive fields of both were within 0.2°) for Monkeys 1 and 2, respectively.

**Figure 5:**
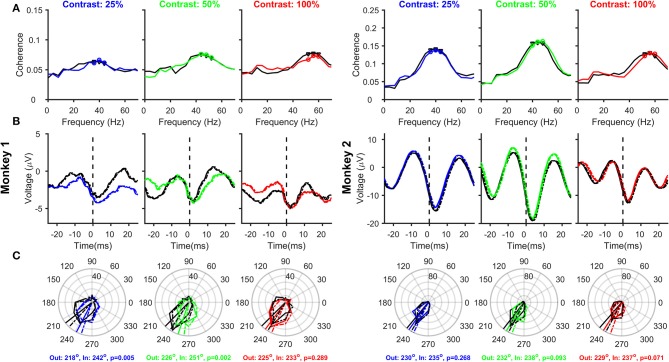
Relationship between Spikes and LFPs recorded from different electrodes, as a function of Contrast and Attention, after removing the filtering effect. Same as Figure 4, but after removing the effect of the low-pass filter on the LFP (solid traces). The stLFP and spike-gamma phase histogram plots in Figures 4B,C are overlaid as dashed-dot traces on the corresponding panels to show the outcome of filtering-effect removal on these measures.

Any causal filter necessarily introduces a delay in the signal, which may be dependent on the frequency of the signal. Butterworth filters have a linear relationship between phase delay and frequency, such that the group delay (which roughly translates to how much each frequency component of the signal shifts in time due to the filtering process) is almost constant over a wide frequency range. We removed the low-pass filtering effect by dividing the Fourier Transform of the LFP by the Fourier Transform of the low-pass LFP filter (4th order Butterworth filter with a low-pass cutoff at 500 Hz; constructed in MATLAB using the command “butter”) and subsequently taking the inverse Fourier Transform (Okun, 2017). The correction was only done between 0 and 500 Hz because the power of the LFP (as well as the filter) was very less beyond 500 Hz. The group delay of this Butterworth filter was ~0.8 ms over almost the entire frequency range of interest (including the gamma range), such that the stLFP constructed from the corrected LFP signal had a similar shape as the uncorrected stLFP but was shifted leftward by ~0.8 ms (**Figure 5B** vs. Figure 4B).

Note that in addition to this low-pass filter, three other filtering operations also need to be accounted for. The data acquisition system had two analog hardware filters: a high pass filter at 0.3 Hz (first order, Butterworth) and a low-pass filter at 7,500 Hz (third order, Butterworth). In addition, to obtain spike data, the signal was high-pass filtered at 250 Hz (fourth order, Butterworth, digital). However, all three filters had negligible group delay (<0.1 ms) between 500–5,000 Hz, suggesting that these filtering operations did not change the position of the spike appreciably. Similarly, the high-pass filter had a large group delay at very low frequencies, as shown by Okun (2017), but it was negligible in the gamma range. Therefore, these three filters did not have an appreciable effect on the spike-gamma phase estimation.

## Results

### Spatial attention reduces alpha and gamma power and increases peak gamma frequency in area V1

We first analyzed changes in alpha and gamma power and gamma peak frequency for attend-in versus attend-out conditions to test whether our results were in agreement with previous attention studies in macaque primary visual cortex (Chalk et al., 2010). Figure 2A shows the average change in PSD during the stimulus period (150–400 ms after stimulus onset) from the pre-stimulus baseline (−300 to −50 ms), for attend-out (black trace) and attend-in condition (color trace) for 25% (blue), 50% (green), and 100% (red) contrasts. Gamma peak frequency increased with increasing contrast (traces for different contrasts are overlaid in the rightmost plot for comparison), as reported previously (Ray and Maunsell, 2010). To account for potential differences in the baseline activity due to attention, all changes were computed with respect to the baseline activity of the unattended condition (see section Materials and Methods). Consistent with previous studies, we found a strong suppression of alpha power due to attention in both monkeys, which could be observed in the baseline PSD as well (dark gray trace), confirming that the monkeys were indeed attending to the stimuli.

To test these results quantitatively, we first performed a three-way ANOVA test with factors of monkey (2 levels: Monkey 1 and 2), attention (attend-out, attend-in) and contrast (25, 50, and 100%). Alpha power was averaged over 8 and 12 Hz (inverted triangles in Figure 2A), while gamma power was averaged in an eight Hz band around the peak frequency for each contrast (40, 48, and 56 Hz for Monkey 1, and 40, 44, and 56 Hz for Monkey 2; Figure 2A; see section Materials and Methods for details). The factor monkey was significant for alpha power (*F*_monkey_ = 243.23, *p* = 9.3 × 10^−49^), gamma power (*F*_monkey_ = 417.45, *p* = 1.3 × 10^−76^), and peak gamma frequency (*F*_monkey_ = 4.52, *p* = 0.004). Thus, we performed a two-way ANOVA with factors attention and contrast separately for the two monkeys for alpha power, gamma power and peak gamma frequency. The effect of contrast on alpha power was not significant for Monkey 1 (*F*_contrast_ = 0.65, *p* = 0.522) but significant effect for Monkey 2 (*F*_contrast_ = 7.06, *p* = 9 × 10^−4^). The effect of attention on alpha power was significant for both monkeys (*F*_attention_ = 24.88, *p* = 9.4 × 10^−7^ for Monkey 1 and *F*_attention_ = 10.17, *p* = 1.5 × 10^−3^ for Monkey 2). However, there was no significant interaction between the two factors on alpha power for either monkey (*F*_contrast × attention_ = 0.87, *p* = 0.42 for Monkey 1 and *F*_contrast × attention_ = 0.91, *p* = 0.40 for Monkey 2). For gamma power, the effect of contrast was significant for both monkeys (*F*_contrast_ = 11.9, *p* = 9.8 × 10^−6^ for Monkey 1 and *F*_contrast_ = 37.78, *p* = 4.6 × 10^−16^ for Monkey 2) but the effect of attention was not significant (*F*_attention_ = 0.3, *p* = 0.58 for Monkey 1 and *F*_attention_ = 1.72, *p* = 0.19 for Monkey 2). Again, there was no interaction between the factors (*F*_contrast × attention_ = 0.05, *p* = 0.95 for Monkey 1 and *F*_contrast × attention_ = 0.17, *p* = 0.85 for Monkey 2). For peak gamma frequency, there was significant effect of both contrast and attention in both monkeys (*F*_contrast_ = 415.46, *p* = 1.6 × 10^−95^ for Monkey 1 and *F*_contrast_ = 973.36, *p* = 7.7 × 10^−178^ for Monkey 2; *F*_attention_ = 5.07, *p* = 0.025 for Monkey 1 and *F*_attention_ = 33.31, *p* = 1.4 × 10^−8^ for Monkey 2). The interaction of the two factors was significant only in Monkey 2 (*F*_contrast × attention_ = 1.11, *p* = 0.33 for Monkey 1 and *F*_contrast × attention_ = 3.37, *p* = 0.04 for Monkey 2). Similar results were obtained in a 2-factor ANOVA performed on the data pooled over the two monkeys.

These results were further confirmed using pairwise t-tests. In almost all conditions, alpha power significantly reduced with attention (Figure 2B; Monkey 1: *t*-test, *N* = 63, *p* = 3.9 × 10^−7^, 1.1 × 10^−13^, 3.5 × 10^−5^ for 25, 50, and 100% contrasts respectively; *p* = 8.9 × 10^−22^ for all the contrast conditions combined; Monkey 2: *t*-test, *N* = 89, *p* = 0.057, 2.4 × 10^−14^, 3.1 × 10^−13^ and 8.6 × 10^−20^ for 25, 50, 100% contrasts and the combined condition, respectively). The reduction in gamma power was significant only for the 50% contrast condition for Monkey 1 (*t*-test, *N* = 63, *p* = 0.41, 0.013, 0.86, and 0.052 for 25, 50, 100% and combined contrast conditions, respectively), and for 25 and 100% contrasts for Monkey 2 (*t*-test, *N* = 89, *p* = 3.4 × 10^−8^, 0.72, 0.014 and 1.5 × 10^−6^ for 25, 50, 100% and combined contrast conditions, respectively). Similarly, the increase in gamma peak frequency was significant only for 25% contrast condition for Monkey 1 (*t*-test, *N* = 63, *p* = 0.007, 0.15, 0.13, and 7.8 × 10^−4^ for 25, 50, 100% and combined contrast conditions, respectively), and all contrasts for Monkey 2 (*t*-test, *N* = 89, *p* = 1.7 × 10^−9^, 6.7 × 10^−11^, 0.013 and 6.7 × 10^−19^ for 25, 50, 100% and combined contrast conditions, respectively).

The weak effect of attention on gamma power is not surprising for two reasons. First, because we recorded from a chronically implanted microelectrode array, the stimuli were optimized only for a single site in each session and therefore were non-optimal for most electrodes, unlike the study by Chalk et al. (2010) where stimuli were better optimized. Second, since the stimuli were only presented for 400 ms (to minimize attentional fluctuations within the stimulus duration) and the analysis duration was only 250 ms, the frequency resolution was 4 Hz, which made it difficult to correctly estimate peak frequency shifts that are typically only 2–3 Hz in V1 (Ray and Maunsell, 2010; Bosman et al., 2012). Note that the second limitation can be partially overcome by using Matching Pursuit (Chandran et al., 2016), which allowed us to better characterize the gamma peak frequency shifts in a previous study (see Supplementary Figure 2 of Ray and Maunsell, 2010); we have used multi-taper analysis here because the spike-field coherence (SFC), which was also used to get spike-gamma phase, was obtained using the same technique. In general, the effects of attention on V1 were consistent with the findings of Chalk et al. (2010), and were almost always significant when the results were pooled across contrasts.

### Effect of contrast and attention on SFC, stLFP, and spike-gamma phase computed using spike and LFP recorded from the same electrode

Next, we analyzed how attention modulated SFC, stLFP and spike-gamma phase when spikes and LFP were recorded from the same electrode (23 and 39 sites for the two monkeys; see Materials and Methods for details), as was the case in the study by Chalk et al. (2010). The magnitude of the SFC (Figure 3A) showed clear peaks in the gamma frequency range, and the peak gamma frequency shifted with an increase in contrast. Consistent with Chalk et al. (2010) and the results obtained using power (Figure 2), we found a reduction in SFC magnitude and an increase in peak gamma frequency with attention in almost all conditions. The stLFP plots (Figure 3B) showed the presence of a prominent rhythm around the time of the spike, especially for Monkey 2, whose trough was shifted 3–4 ms away from zero. These results were reflected in the spike-gamma phase histograms (Figure 3C), obtained by taking the average angle of the SFC across the three frequency bins around the peak gamma frequency (as highlighted in Figure 3A). Following the convention used by Chalk and colleagues, phase angles were defined such that trough of the gamma rhythm was at 180° and rightward shift of the trough increased the phase angle. The mean phase angles were ~210° for Monkey 1 and ~235° for Monkey 2 and were not significantly different across attention conditions (circular mean phases and the associated p-values obtained from Watson-Williams test are shown in the legend). Even when pooled across contrasts, the mean phases between attend-in and attend-out conditions were not significantly different (Watson-Williams multi-sample test, *p* = 0.28 and 0.33 for Monkeys 1 and 2). Similarly, the mean phases at different contrasts were not significantly different from each other in either attend-out or attend-in conditions (Watson-Williams multi-sample test, *p* = 0.89 (attend-out) and *p* = 0.9 (attend-in) for Monkey 1; *p* = 0.66 (attend-out) and *p* = 0.55 (attend-in) for Monkey 2). These results are consistent with Chalk et al. (2010), who obtained a median phase of ~-0.65π, which translates to ~243°. An offset of ~30°-50° from the trough (180°) is also consistent with the findings of Vinck et al. (2010) and Ray and Maunsell (2010), although in these two studies the convention was chosen such that rightward shift of the trough led to a reduction of phase angle below 180° (such that the phase angles were between ~130° and ~150°).

Although our results are consistent with previous studies, there are two serious flaws in these results, which can be clearly observed in the stLFP plots (Figure 3B). First, there is a large spike-related transient (sharp negative dip near time zero), which biases the estimation of the gamma phase. Specifically, this transient can be decomposed into a series of sinusoids with their troughs aligned to the trough of the transient, effectively “pulling” the phase of any true phase-locked rhythm toward 180° (for a detailed discussion, see Ray, 2015). This can be observed in the two monkeys: the estimated spike-gamma phase is closer to 180° for Monkey 1 compared to Monkey 2 (~210° vs. ~235°), simply because the relative magnitude of the transient compared to the gamma rhythm is larger for Monkey 1. The second flaw is that the spike-related transient, which should be around the time of the spike itself, is shifted toward positive values. We address both these concerns below.

### Effect of using different electrodes for spikes and LFP on SFC, stLFP, and spike-gamma phase

One popular method to reduce the spike-related transient is to take spikes and LFP from different electrodes (Ray and Maunsell, 2010; Vinck et al., 2010; Ray, 2015). We, therefore, repeated the analysis on 163 and 170 “different” spike-LFP pairs for the two monkeys, such that the receptive fields of both were located within 0.2° of stimulus center (see section Materials and Methods for details). Mean SFC showed similar results as before, with clear peaks in the gamma frequency range and an increase in peak gamma frequency with increasing contrast, and a slight reduction in SFC magnitude and an increase in peak frequency with attention in some cases. Spike-related transient, which was prominent in Figure 3B, was now much reduced, better revealing the true gamma rhythm in the stLFP (Figure 4B) whose trough was 3–4 ms after the spike in both the monkeys. Mean spike-gamma phases were now ~235° and ~245° for the two monkeys (Figure 4C; note that the shift in mean phase between Figures 3, 4 is much larger for Monkey 1 because the spike transient was relatively much larger for that monkey). Interestingly, for both monkeys and for all contrast conditions, attention appeared to shift the mean gamma-spike phase away from 180°. Although this phase difference did not reach significance for many contrast levels (circular means and p-values obtained using Watson-Williams multi-sample test are shown in the bottom of Figure 4C), the phase differences were highly significant when combined across contrasts (Watson-Williams multi-sample test, *p* = 7 × 10^−5^ and 5 × 10^−3^ for Monkeys 1and 2), albeit the actual magnitude of the difference was small (~19° and ~6°). The mean phases at different contrasts were not significantly different from each other in either the attend-out or the attend-in condition [Watson-Williams multi-sample test, *p* = 0.36 (attend-out), *p* = 0.11 (attend-in) for Monkey 1 and *p* = 0.7 (attend-out), *p* = 0.34 (attend-in) for Monkey 2].

### Effect of removing the filtering artifact on SFC, stLFP, and spike-gamma phase relation

The rightward shift of the spike-related transient away from zero (Figures 3B, 4B) is simply due to the effect of the filtering operation to obtain the LFP. We, therefore, removed this filtering effect (see Materials and Methods for details) and reanalyzed SFC, stLFP and spike-gamma phase for “different” pair condition (Figure 5; for the “same” electrode condition, this operation caused the trough of the spike-transient to shift near zero; data not shown). While this operation did not change any of the results shown in Figure 4, the mean phases decreased by ~10° at both 25 and 50% and ~13° at 100% contrast (for the same shift in time, the shift in degrees depends on the frequency of the rhythm; Figures 4B,C are overlaid as dashed-dot traces on the corresponding panels in Figure 5 to show the outcome of filtering-effect removal). Otherwise, like Figure 4, the effect of attention on spike-gamma phase remained significant when phases were pooled across contrast conditions (Watson-Williams multi-sample test, *p* = 6 × 10^−5^ for Monkey 1 and 8 × 10^−3^ for Monkey 2). Similarly, the mean phases at different contrasts were not significantly different from each other in either the attend-out or the attend-in condition [Watson-Williams multi-sample test, *p* = 0.58 (attend-out), *p* = 0.07 (attend-in) for Monkey 1 and *p* = 0.69 (attend-out), *p* = 0.73 (attend-in) for Monkey 2].

## Discussion

We investigated whether increasing stimulus contrast or allocating more attention to a stimulus (which increases its effective contrast) shifts the position of the spike relative to the phase of the gamma rhythm, as posited by the PC hypothesis. We highlighted two issues that can bias the phase estimation: the presence of the spike-related transient and the effect of filtering to obtain the LFP. After accounting for these issues, we found no effect of stimulus contrast and a weak but significant effect of attention on spike-gamma phase. Although these results are consistent with the PC hypothesis in the context of attention, we discuss three issues that severely limit the efficacy of gamma PC in V1.

### Issue 1: magnitude of gamma PC in V1

For a rhythm occurring at 50 Hz (time period of 20 ms), the interval between the peak and the subsequent trough (the interval over which the inhibition fades away) is 10 ms, which is the maximum range over which PC can operate. It is clear from our results, as well as prior reports, that even if PC occurs, it only uses a small sub-interval within this interval. Since spikes occur away from the trough of the rhythm with increasing stimulus intensity under PC, the delay of the trough from the spike at 100% contrast sets the dynamic range of this coding scheme. In our data, spikes occurred at ~230° at 100% contrast, similar to the value reported by Chalk et al. (2010) (~240°) and Vinck et al. (2010) (~137° for preferred orientation, which translates to ~223° as per our convention). For a rhythm at ~50 Hz, a shift of ~50° translates to only ~3 ms out of the available ~10 ms for coding. Further, even when contrast was reduced to 25%, there was no discernable change in the trough position. The only study that did show any evidence of phase coding (Vinck et al., 2010) showed a shift of ~20° between the best and worst orientation, which translates to only ~1 ms shift (in addition, see other issues with their results below). In our data, the shift in phase due to attention is even lesser, especially for Monkey 2 (in addition, see Issue 3 below). It can be argued that the phase could shift down to 180° for very low contrasts (providing a dynamic range of ~3 ms), but it is well known that gamma rhythm itself is weak or absent at very low contrasts (Henrie and Shapley, 2005; Jia et al., 2013) and also peaks at a lower frequency (see Issue 3). Thus, if we consider the range of contrasts for which gamma is reliable, the magnitude of PC (i.e., the range over which the spike varies with respect to the rhythm) appears to be very small in V1. In this context, our filtering correction becomes significant, since even though the group delay is only ~0.8 ms, it still decreases the dynamics range by a further ~20–25%.

### Issue 2: effect of changing gamma amplitude

As shown in Figure 3, spikes are associated with a transient in the LFP recorded from the same electrode, which biases the spike-LFP phase analysis. Because the amplitude of an extracellular action potential generally decreases rapidly as a microelectrode is moved away from the neuron (Gold et al., 2006; Schomburg et al., 2012), the spatial spread of a spike is thought to be very local (for example, Xing and colleagues used a range between 30 and 100 μm for single units; Xing et al., 2009). Therefore, one way to reduce the spike transient is to take the LFP from a neighboring electrode that is separated from the spike electrode by at least a few hundred microns (for a representative case, see Vinck et al., 2010). There are, however, two issues with this approach. First, although taking spikes and LFPs from different electrodes drastically reduces the spike-related transient, it does not completely eliminate it (Ray, 2015). For example, as shown in Figures 2A,E of Ray and Maunsell (2011b) where stLFPs were constructed using spikes and LFP electrodes separated by different distances for the same two monkeys as used in this study, the spike-transient could be seen up to electrode pairs separated by ~400 μm for Monkey 1 and ~0.4–1.6 mm for Monkey 2, albeit the magnitude of the spike-transient was much smaller than when stLFP was constructed from the same electrode (*d* = 0 condition in those plots). This happens because neurons near the LFP electrode are often correlated with the neuron being recorded from the spike electrode, and those neurons produce a transient in the LFP electrode that are locked to the spikes on the spike electrode. The second issue is that this procedure implicitly assumes that gamma oscillations recorded from two nearby electrodes are similar, but the spatial spread of LFP itself is a topic of debate. While some studies have shown that the spatial spread of LFP could be large (up to a few mm; Kajikawa and Schroeder, 2011), others have shown that it could be only a few hundred microns (Katzner et al., 2009; Xing et al., 2009; Dubey and Ray, 2016). A modeling study showed that the spread could depend on the degree of correlation in the neural population (Lindén et al., 2011). Consequently, there might be differences in the gamma recorded from neighboring microelectrodes. For example, we have shown that when a Gabor stimulus is presented, two microelectrodes separated by as little as 0.2° can exhibit significantly different center frequencies (Ray and Maunsell, 2010). Therefore, some studies have used other techniques to remove the spike-transient, such as Matching Pursuit (Ray et al., 2008) or a Bayesian Framework (Zanos et al., 2011). All these methods substantially reduce the spike-transient, although it is unlikely that they completely eliminate it.

A small spike-transient is unlikely to influence the estimation of gamma phase when the rhythm itself is very strong but may shift the phase toward 180° when the rhythm is weak. For example, even when spikes and LFPs were recorded from separate electrodes (Figure 4), the mean phases for Monkey 1 were about ~10° less than Monkey 2, who had a much stronger gamma rhythm than Monkey 1. A visual inspection of the stLFP (Figure 4B) reveals a small spike-transient like structure in Monkey 1, which could have contributed to the reduction in spike-gamma phase as compared to Monkey 2. Importantly, in cases where the magnitude of gamma itself varies across conditions, an apparent shift in spike-gamma phase could just be due to a differential contribution of the spike-transient which “pulls” the phase toward 180°. For example, Vinck et al. (2010) showed that gamma PC was stronger when gamma power and gamma phase locking was very weak (see their Figure 6). Because they did not show the stLFPs, it is unclear whether the apparent phase shift they documented was because of a genuine leftward shift of the gamma trough or the presence of a spike-transient whose contribution was larger when the gamma rhythm itself was weak.

### Issue 3: effect of changing gamma peak frequency

PC hypothesis makes sense when the rhythm has a stable frequency. However, the center frequency of gamma rhythm varies systematically with changes in a variety of stimulus parameters, such as size (Gieselmann and Thiele, 2008; Ray and Maunsell, 2011a; Jia et al., 2013), contrast (Ray and Maunsell, 2010; Bosman et al., 2012; Jia et al., 2013), and drift rates (Gray and Viana Di Prisco, 1997; Friedman-Hill et al., 2000). For example, although we show that the spike-gamma phase angles do not vary with stimulus contrast, note that these angles are computed for different gamma frequencies, making it harder to interpret and compare these phase values. Vinck et al. (2010) used different orientations for comparison, but gamma center frequencies can vary even for different orientations, although the trends are not always consistent (see Figure 2D of Jia et al., 2013 and Figure 1 of Murty et al., 2018). For the same delay between the spike and gamma trough, the effective phase angle is greater when the rhythm is faster. For example, in our data, the stLFP troughs appear to coincide between the attend-in and attend-out cases in almost all conditions (Figure 5B). However, since attention slightly increases the gamma frequency, the effective phase lag in degrees could be larger, which could explain the small but consistent increase in phase angles.

We note, however, that we computed phase over a 250 ms window (similar results were obtained for 200 ms window), which cover more than 10 cycles of the rhythm. During natural vision, we make 3–4 saccades every second (even during fixation, we make several micro-saccades per second), and such eye movements can change or reset the phase of LFP oscillations (Bosman et al., 2009; Ito et al., 2011). It is possible that PC occurs within a single or a few cycles of gamma rhythm, for which gamma need not even have stable frequency over time. It is also possible that PC occurs differently during natural viewing as opposed to a paradigm where animals are trained to fixate for long durations. For example, Ito and colleagues showed that in freely viewing monkeys, fixation-related spike synchronization occurred at an early phase of the rate response after fixation-onset, and the first spikes after the onset of a fixation were locked to a specific epoch of the LFP modulation (Ito et al., 2011). Other studies have also shown that gamma rhythm tends to appear in short bursts over a few cycles (Xing et al., 2012; Lundqvist et al., 2016; Chandran Ks et al., 2017), and therefore PC could theoretically occur over shorter duration than what was considered here. Comparable recordings from monkeys during natural viewing conditions as well as advanced signal processing techniques are required to test this hypothesis.

### Weak effect of attention in V1

The effect of attention was weaker in our data than the findings of Chalk et al. (2010), possibly due to the use of sub-optimal stimuli for many sites, fewer sites, and a shorter analysis window. However, it is unlikely that our results would change drastically if these limitations could be overcome. First, the effect of attention on gamma in V1 is in general weak (Chalk et al., 2010; Buffalo et al., 2011). Second, although the reduction in gamma power and SFC with attention were small, we obtained a pronounced reduction in alpha power in all cases. Similarly, the increase in gamma peak frequency (1–3 Hz in our data) was comparable to a previous study by Bosman and colleagues, who reported an increase in gamma peak frequency of 2–3 Hz (Bosman et al., 2012). Third, although the analysis window was shorter than previous studies, which yielded a poor frequency resolution, the stLFP plots were computed in the time-domain itself and therefore did not suffer from the poor frequency resolution, but even these did not show a substantial rightward shift as is expected from the PC hypothesis. Fourth, while we had fewer recording sites that may have yielded less statistical power for power analysis (Figure 2), we had a substantial number of pairs (163 and 170 for the two monkeys), so the main result regarding the PC hypothesis (Figure 5) did not suffer from the lack of statistical power. Finally, although the effect of attention was weak, contrast had a strong effect on gamma power and frequency, but the PC hypothesis for contrast did not yield a significant result.

In summary, although we did find a weak effect of attention on spike-gamma phase relationship, based on the variety of issues that we have discussed, gamma PC is at best expected to play a minor role in the coding the stimulus contrast in V1.

## Author contributions

AD and SR conceived the idea of research. SR collected data; AD and SR analyzed data. AD and SR wrote the paper.

### Conflict of interest statement

The authors declare that the research was conducted in the absence of any commercial or financial relationships that could be construed as a potential conflict of interest.
